# Outcomes of Resectable Gallbladder Cancer: A Retrospective Analysis From a Tertiary Care Centre in India

**DOI:** 10.7759/cureus.65735

**Published:** 2024-07-30

**Authors:** Sanjay Chandra Das, Joydeep Ghosh, Sagar Kanta, Harsh Thakran, Sandip Ganguly, Bivas Biswas, Sudeep Banerjee, Manas Kumar Roy, Tanuj Chawala

**Affiliations:** 1 Department of Emergency Medicine, Tata Medical Center, Kolkata, IND; 2 Department of Medical Oncology, Tata Medical Center, Kolkata, IND; 3 Department of Gastrointestinal (GI) and Hepatopancreatobiliary (HPB) Surgery, Tata Medical Center, Kolkata, IND

**Keywords:** resectable gallbladder cancer, radical cholecystectomy, overall survival (os), disease-free survival (dfs), adjuvant chemotheapy

## Abstract

Background

Gallbladder carcinoma (GBC) has deleterious outcomes, but due to its reduced incidence in Western countries, there is a paucity of data on this disease. Here we report the outcomes of a retrospective analysis of resectable gallbladder cancer from a tertiary cancer centre in eastern India. The primary objective of this study is to evaluate the overall survival (OS) and relapse-free survival (RFS) rates among patients with resectable GBC.

Methods

A retrospective analysis was carried out on patients who underwent radical surgery between 2007 and 2022 and received various neoadjuvant and adjuvant chemotherapy methods. Patients who had adjuvant chemoradiotherapy concurrently or who did not receive adjuvant therapy were excluded. All the baseline clinicopathological characteristics were retrieved from electronic medical records. The survival data were collected from records of follow-up visits as well as telephonic calls to the patients who were lost to follow-up. Simple proportions were used for baseline characteristics, and the Kaplan-Meier method was used for survival analysis.

Results

A total of 161 patients were identified, and data were captured from electronic medical records. The included patients' ages ranged between 26 and 80 years, with a median age of 56 years. Among the participants, 103 were female (64%) and 58 (36%) were male. Among the 161 patients, the median number of lymph nodes harvested was nine (ranging from one to 43), and only three patients were margin-positive. The tumour, nodes, and metastasis (TNM) distributions were as follows: pT2 in 111 patients (70.25%), pT3 in 44 patients (27.85%), and pT4 in three patients (1.90%). The nodal statuses were pN0 in 91 patients (61.9%), pN1 in 51 patients (34.69%), and pN2 in five patients (3.4%).

The majority (64%) received single-agent capecitabine, 27% received gemcitabine-based platinum doublet therapy, and 4.3% received neoadjuvant therapy. Of the full sample, 2.4% received concurrent adjuvant chemo plus radiation therapy, and three patients did not receive any adjuvant therapy. Additionally, among the 161 patients, 34.16% had a relapse, with 47% being local and 52% being distant relapses. The median follow-up was 49 months (interquartile range (IQR) 23-71 months). The 24-month RFS rate was 67.1% (SD+/- 4.3%), and the 24-month OS rate was 78.1% (SD+/- 4.1%).

Conclusion

Our data, which is from one of the largest samples from India, show that resectable gallbladder cancer has very good outcomes after radical surgery and adjuvant chemotherapy. There was a higher proportion of T2 and node-negative disease, which could have led to better survival compared to published literature.

## Introduction

Gallbladder carcinoma (GBC) is not an uncommon cancer. More than 115,000 new cases of GBC were diagnosed in 2020, with the majority reported in Asia, the continent responsible for 0.9% of global cancer-related deaths [1]. In India, there is regional variation in GBC prevalence, with higher incidence in the northern, north-eastern, central, and eastern parts of India, probably owing to the predominant diet and the presence of pollution in the river Ganga [2, 3].

The treatment of localized gallbladder cancer involves surgery followed by adjuvant capecitabine. The BILCAP trial has shown that adjuvant capecitabine improves overall survival compared to observation [4]. Other trials that used gemcitabine-based regimens have failed to show any significant benefit [5,6]. Thus, at present, the standard of care in resected gallbladder cancer is the administration of capecitabine. There have been attempts to evaluate the role of adjuvant chemoradiotherapy in resected gallbladder cancer, but most such trials were single-arm studies, with no study detailing a comparison between chemotherapy and radiotherapy. A randomized phase 2 trial is ongoing at Tata Memorial, Mumbai, India, to compare adjuvant chemotherapy with chemoradiation, but the results are awaited [7].

Most of the above trials have been conducted in Western countries, where the incidence of gallbladder cancer is quite low. By contrast, India has a very high incidence of GBC, implying the importance of real-world evidence of adjuvant therapy. This study thus analyzes adjuvant treatment data from a large-volume tertiary cancer centre from the western part of the country, which is a high-incidence area.

The primary objective of this study is to evaluate the overall survival (OS) and relapse-free survival (RFS) rates among patients with resectable GBC. The study also focuses on assessing and describing the clinical, pathological, and treatment-related aspects of resectable GBC. We examine the results of resectable GBC patients in an effort to offer insightful information on the prognosis and treatment of this cancer with the ultimate goal of enhancing patient care, therapeutic approaches, and overall survival outcomes in India.

This article was previously presented as a poster at the European Society for Medical Oncology (ESMO) World Congress on Gastrointestinal Cancer on 29 June 2023.

## Materials and methods

A retrospective analysis was carried out on patients who underwent radical surgery between 2007 and 2022 and received various neoadjuvant and adjuvant chemotherapy methods at Tata Medical Center, Kolkata, India. Patients who had adjuvant chemoradiotherapy concurrently or who did not receive adjuvant therapy were excluded. The data for our analysis were sourced from the electronic medical records of a total of 161 patients who underwent radical cholecystectomy and received various modalities of therapy. We examined several key variables, including age, gender, date of diagnosis, staging of the tumour, date of radical surgery, start date and end date of adjuvant chemotherapy, date and site of relapse, survival status, and date of the last follow-up.

All the baseline clinicopathological characteristics were retrieved from electronic medical records. The survival data were collected from records of follow-up visits as well as telephonic calls to the patients who were lost to follow-up. Subsequently, the collected data in simple proportions were used for baseline characteristics, while the Kaplan-Meier method was employed to analyze the RFS and OS rates. Since this is a retrospective study of electronic medical records that did not involve patients directly, a consent waiver was obtained from the institutional review board of Tata Medical Center.

## Results

The included patients' ages ranged between 26 and 80 years, with a median age of 56 years. Among the participants, 103 were female (64%) and 58 (36%) were male. Data obtained from the number staging system indicate that most of the patients reported stage 2 and stage 3 cases. While one case reported stage ypTis (received neoadjuvant chemotherapy with complete response), 50.31% of patients had stage 2, 45.96% had stage 3, and 3.1% had stage 4 cancer (Table 1). The histopathological distribution of our sample is as follows: adenocarcinoma in 148 cases (91.9%), adenosquamous carcinoma in four cases (2.4%), papillary carcinoma in four cases (2.4%), squamous cell carcinoma in two cases (1.2%), signet cell carcinoma in two cases (1.2%), and small cell carcinoma in one case (Table 2).

**Table 1:** Stages of cancer ypTis: received neoadjuvant chemotherapy with complete response

Stage	Number of patients
ypTis	1
1	0
2	81
3	74
4	5

**Table 2:** Histopathological characteristics of the study group

Types	Total number of cases
Adenocarcinoma	148
Adenosquamous carcinoma	4
Papillary carcinoma	4
Squamous cell carcinoma	2
Signet cell carcinoma	2
Small cell carcinoma	1

Among the 161 patients, the median number of lymph nodes harvested was nine (ranging from one to 43), and only three patients were margin-positive. The tumour, nodes, and metastasis (TNM) distributions were as follows: pT2 in 111 patients (70.25%), pT3 in 44 patients (27.85%), and pT4 in three patients (1.90%). The nodal statuses were pN0 in 91 patients (61.9%), pN1 in 51 patients (34.69%), and pN2 in five patients (3.4%).

Out of the 161 patients, 64% received capecitabine, 27% received gemcitabine-based platinum doublet therapy, and 4.3% received neoadjuvant therapy. Of the full sample, 2.4% received concurrent adjuvant chemo plus radiation therapy, and three patients did not receive any adjuvant therapy. Additionally, among the 161 patients, 34% had a relapse, with 47.2% (n = 26) being local and 52.7% (n = 29) being distant relapses. Among patients who received capecitabine alone, 26.2% had a relapse, and 56% of them were metastatic. Further, 53.4% of patients who received platinum doublet therapy had a relapse, and 48% of them were metastatic. A total of seven patients received neoadjuvant chemotherapy. After surgery, three of these patients did not receive adjuvant therapy, while the remaining four did. The neoadjuvant therapies administered included gemcitabine-cisplatin, gemcitabine-carboplatin, cisplatin-etoposide, and gemcitabine-oxaliplatin (Table 3). Among the patients who received neoadjuvant therapy before surgery, 28.6% had a relapse (n = 2); one was metastatic, and the other was local. Only one patient out of the four who received concurrent chemoradiation therapy had a relapse, and it was metastatic. Out of the three patients who did not receive any adjuvant therapy, one had a metastatic relapse (Table 4).

**Table 3:** Neoadjuvant chemotherapy GEM: gemcitabine; CIS: cisplatin; ETO: etoposide; CARBO: carboplatin; OX: oxaliplatin

Neoadjuvant chemotherapy	Adjuvant chemotherapy
GEM-CIS	Continued GEM-CIS
GEM-CIS	Continued GEM-CIS
GEM-CIS	Not continued
GEM-CIS	Not continued
CIS-ETO	Not continued
GEM-CARBO	Continued GEM-CARBO
GEM-OX	Continued GEM-OX

**Table 4:** Regimes followed Pt: platinum-based; GEM: gemcitabine; CIS: cisplatin; CARBO: carboplatin; OX: oxaliplatin; CAPE: capecitabine; 5FU: 5 fluorouracil; CETU: cetuximab

Therapy	Number	Relapse	%	Local	%	Metastasis	%
Capecitabine	103	27	26.21	12	44.44	15	55.55
Pt doublet (gem-cis/gem-ox/gem-carbo)	43	23	53.48	12	52.17	11	47.82
Neoadjuvant	7	2	28.57	1	50	1	50
Concurrent adjuvant chemoradiotherapy	4	1	25	0	0	1	25
No adjuvant	3	1	33	0	0	1	33
GEM-CAPE	3	1	33	0	0	1	33
GEM	4	2	50	1	25	1	25
GEM+5FU+CETU	1	0	0	0	0	0	0

**Figure 1:**
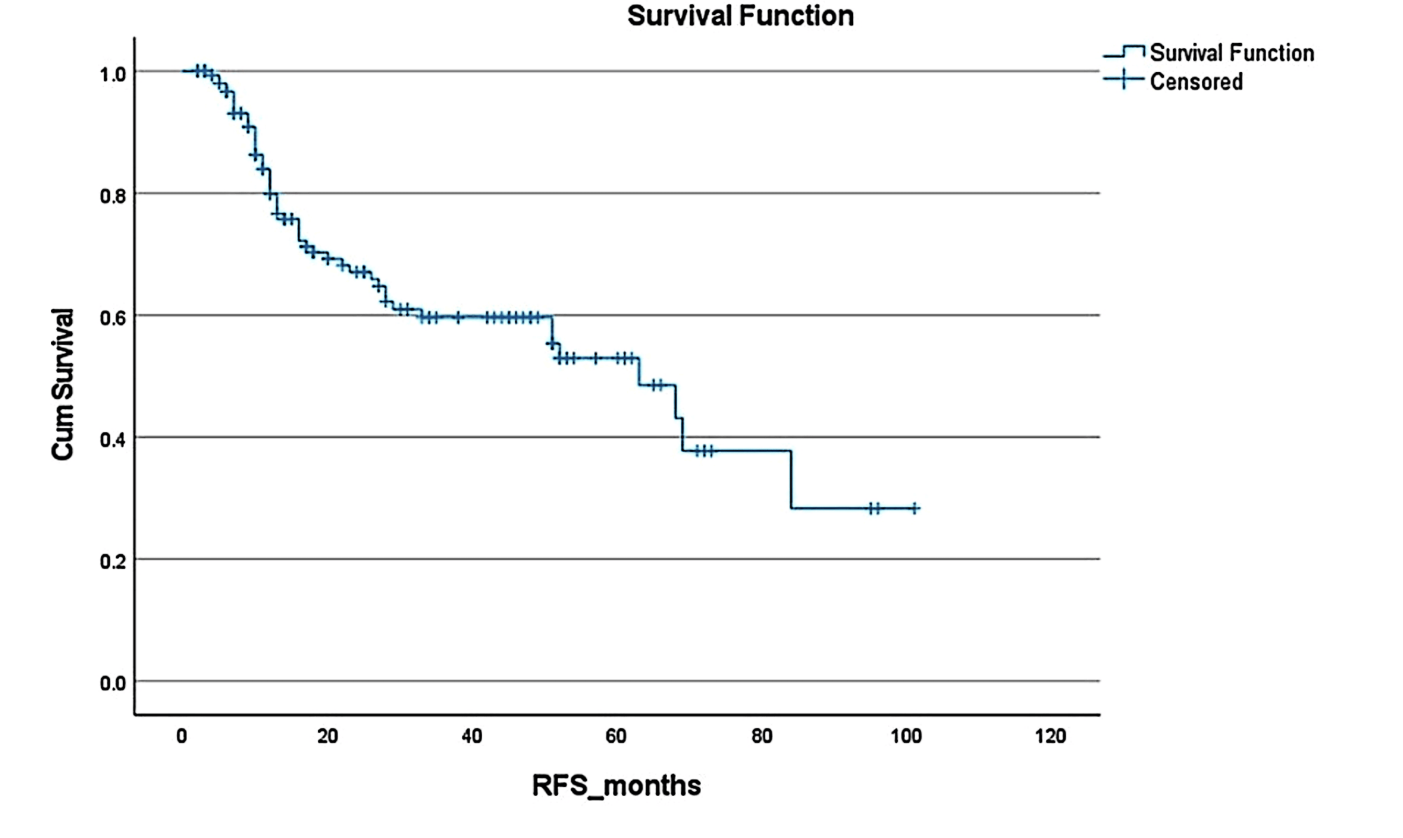
Relapse-free survival (RFS) rate Cum: cumulative

**Figure 2:**
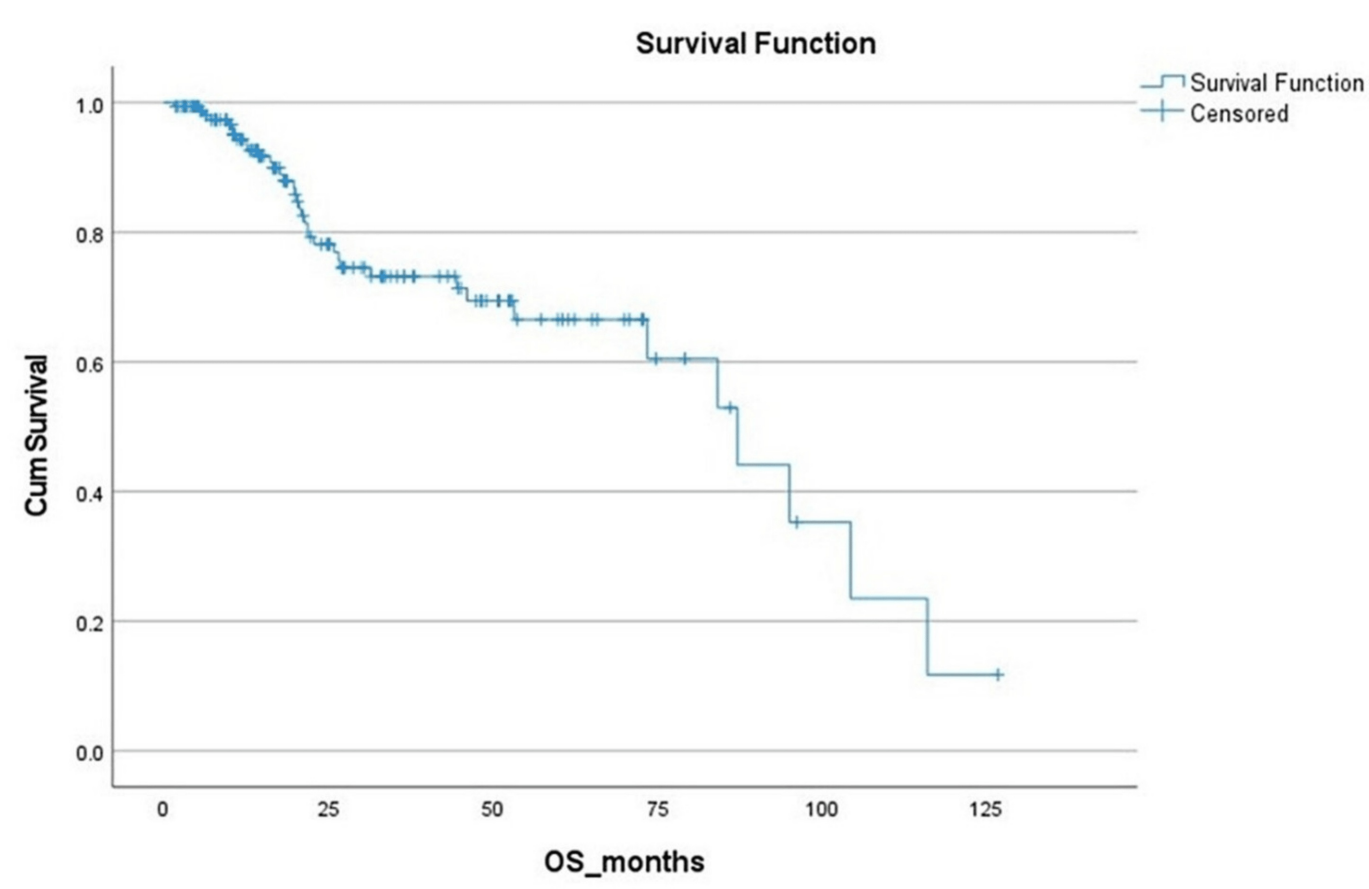
Overall survival (OS) rate Cum: cumulative

The median follow-up was 49 months (interquartile range (IQR) 23-71 months). The 24-month RFS rate was 67.1% (+/- 4.3%; Figure 1), and the 24-month OS rate was 78.1% (+/- 4.1%; Figure 2).

## Discussion

Historically, GBC has been treated with surgery alone. The current standard of care consists of en bloc resection of the tumor with lymph nodal dissection, aiming for R0 resection. However, there is a lack of data regarding the effectiveness of adjuvant treatment modalities in GBC cases within the Indian population. The purpose of this study was thus to estimate the incidence and survival rates of GBC at our institution in the period 2007-2022.

While surgery is the major treatment modality, GBC still has a poor prognosis. The survival rates, though still dismal, have improved with adjuvant treatment compared to the cases of patients who received no adjuvant treatment. Gallbladder carcinoma has a dismal prognosis; the disease's five-year survival rate is less than 5%. Yet, it is possible to have a 75% five-year survival rate in cases of early-stage detection [8].

For instance, Takada et al., evaluating the efficacy of adjuvant treatment in various pancreaticobiliary malignancies, including gall bladder, pancreatic, cholangio, and ampullary carcinomas, found that the major initial benefits of adjuvant treatment were concentrated in GBC cases alone. [9]. Their study paved the way for further prospective studies evaluating adjuvant therapies. A subsequent meta-analysis by Horgan et al., which included all biliary tract cancers, showed the benefits of adjuvant therapy to be insignificant; however, this study was limited by an unclear research methodology, as only one randomized controlled trial (RCT) was included [10].

In a more recent meta-analysis published in 2015, Ma et al. evaluated the cases of about 3,200 patients with GBC, finding that adjuvant chemotherapy was beneficial compared to surgery alone. The adjuvant treatment modality was majorly beneficial in the R1 resection, lymph node positive disease, and stage ≥2 cases. The authors also evaluated regional variabilities, finding that the Asian population showed more benefits than the non-Asians in the sample [11].

While multiple regimens have been tried in adjuvant therapy, most have failed to show extra benefits. For instance, single-agent gemcitabine has shown no benefit [12]. The majority of the patients included in our sample received capecitabine. We found that capecitabine showed the maximum benefit, with a relapse occurring in only 26% of patients receiving it. However, Valle et al. showed that gemcitabine with cisplatin was better in advanced GBCs, and the same was applied in the adjuvant therapy [13]. Yet, Saluja et al. found no benefit of adjuvant platinum with gemcitabine [6], correlating with our data, where gemcitabine with platinum doublet showed more than half of the patients (53%) undergoing relapse.

Gemcitabine and oxaliplatin also did not show beneficial results [5]. Only capecitabine from the BILCAP study showed improvement in survival, but the results were not statistically significant enough to treat the affected population [4]. Further, gemcitabine/capecitabine results have been found to be similar to those of gemcitabine/cisplatin [14]. Overall, multiple regimens have been tried at our institute, and the corresponding data have been presented in this paper (Table 1).

Following adjuvant and neoadjuvant treatment for GBC , more than 34.1% of patients reported a recurrence. Of these recurrences, 47% were local, and 52% were distant metastases. Individuals who encountered recurrence after receiving adjuvant therapy fared better than those who did not receive it, even though chemotherapy did not lower the rate of recurrence. At the time of the last follow-up, 62 patients out of our sample of 161 (38.5%) had a recurrence.

Challenges and limitations

The major limitation of the study is that we only included patients from a single clinical site in our sample. Although this helps maintain consistency in diagnostic standards and procedures, the results may not be applicable for patients from diverse geographical regions, healthcare environments, or demographic backgrounds. To improve the generalizability of the findings, more prospective and RCTs must be conducted across different locations and diverse patient groups.

## Conclusions

The data from our sample, which is one of the largest ones from India, show that patients diagnosed with resectable GBC showed very good outcomes after radical surgery and adjuvant chemotherapy. There was a high proportion of T2 and node-negative disease cases, which could have led to better survival outcomes compared to the published literature. The cases of local recurrence were fewer compared to those of distant recurrence, so there is a need for further trials to examine better systemic control.

This study is intended to shed light on the treatment and prognosis of GBC through the analysis of a large data set, which can help enhance clinical approaches and patient care. For the purpose of providing the largest sample size possible, we collected patient data over an extensive period.
